# Simulation‐based training significantly improved confidence and clinical skills of resident doctors in acute diabetes management

**DOI:** 10.1111/dme.70068

**Published:** 2025-06-17

**Authors:** Kalyaani Persad, Aqeelah Khatoon, Aashritha Buchipudi, Amanda Ling Jie Yee, Nevil C. Philip, Angelica Sharma, Akshat Sinha, Sofia Salahuddin, Punith Kempegowda, DEVI collaboration, Rahul Sagu, Josh Banerjee, Maiar Elhariry, Ketan Dhatariya, Gerry Rayman, Mujahid Saeed, Srikanth Bellary, Mohammed Ali Karamat

**Affiliations:** ^1^ Birmingham Medical School, School of Medical Sciences, University of Birmingham Birmingham UK; ^2^ Department of Diabetes and Endocrinology University Hospitals Birmingham NHS Foundation Trust Birmingham UK; ^3^ Norfolk and Norwich University Hospitals NHS Foundation Trust Norwich UK; ^4^ Applied Health Sciences, School of Health Sciences, University of Birmingham Birmingham UK

**Keywords:** clinical diabetes, DKA, Inpatient diabetes, medical management, professional education

## Abstract

**Aims:**

The joint consensus of the American Diabetes Association and the European Association for the Study of Diabetes highlights the need for practical, up‐to‐date training in acute diabetes care. Therefore, this session aimed to develop an applied learning intervention that identifies key gaps in acute diabetes training, assesses participants' change in confidence in managing these scenarios following the intervention and evaluates participants' satisfaction and the intervention's relevance to practice.

**Methods:**

To design the programme, we adopted Kern's six‐step curriculum development framework. Clinical experts were interviewed to assess general needs, and students and resident doctors were interviewed to assess targeted needs. The SIMBA (Simulation via Instant Messaging for Bedside Application) model was adopted to develop cases alongside generative AI and expert input. Pre‐ and post‐session surveys assessed participants' confidence, while the latter also assessed satisfaction and relevance to practice.

**Results:**

33 participants attended the session. 17 participants completed both pre‐ and post‐session surveys and were included in the study. Simulation performance scores averaged 4.0/5 for history taking, interpretation and clinical judgement; 3.9/5 for physical examination and investigations; and 3.6/5 for management. Confidence in managing acute diabetes scenarios improved significantly (pre vs. post: 33.3% vs. 78.4%, *p* < 0.001). 94.1% strongly agreed the cases were relevant, and 82.3% preferred this teaching method over traditional approaches.

**Conclusion:**

The model identified the need for targeted education on physical examination, investigations and management specific to acute diabetes scenarios. Simulation‐based education significantly enhances confidence and is perceived as highly relevant for training in acute diabetes management.

**What's new?**

- Simulation‐based education is increasingly recognised as an effective method for improving clinical curriculum, and practical training in acute diabetes care is needed.
- This study highlights significant gaps in assessment and management skills specific to acute diabetes scenarios amongst diabetes and endocrine doctors in training.
- Targeted simulation‐based education, leveraging generative AI and expert input, is an effective and relevant approach to address these critical gaps in acute diabetes training.

## 1 INTRODUCTION

Management of acute diabetes scenarios represents a critical aspect of healthcare delivery, with significant implications for patient outcomes and system resources.^1^ Healthcare professionals are trained to recognise and initiate interventions for acute diabetes scenarios.^2^ However, there is reduced training for ongoing metabolic management, patient‐specific adjustments and discharge planning for these scenarios.^3^ This gap in care can result in prolonged hospital stays and delayed discharge, adding to bed pressures within the National Health Service (NHS). For patients, extended hospitalisation increases the risk of hospital‐acquired infections, impacts mental well‐being and can erode the quality of life.

Traditionally, medical education for diabetes management has focused on knowledge transfer but often lacks the hands‐on, contextual learning critical for real‐world application in prolonged patient care.^4^ Simulation‐based learning offers a promising solution to these challenges, providing an environment where healthcare providers can emulate both immediate and extended management of acute diabetes without risking patient safety.^5^ However, integrating simulation into medical education faces logistical challenges, including resource demands, variability in institutional support and limited faculty training.^6^

In response to these issues, this study aimed to develop and evaluate a targeted, simulation‐based training session designed to improve the management of acute diabetes scenarios. The specific objectives were:

1. Identify and address gaps in acute diabetes education.
2. Implement and deliver a comprehensive simulation programme for acute diabetes
3. Evaluate program acceptance and effectiveness

## 2 METHODS

**FIGURE 1:**
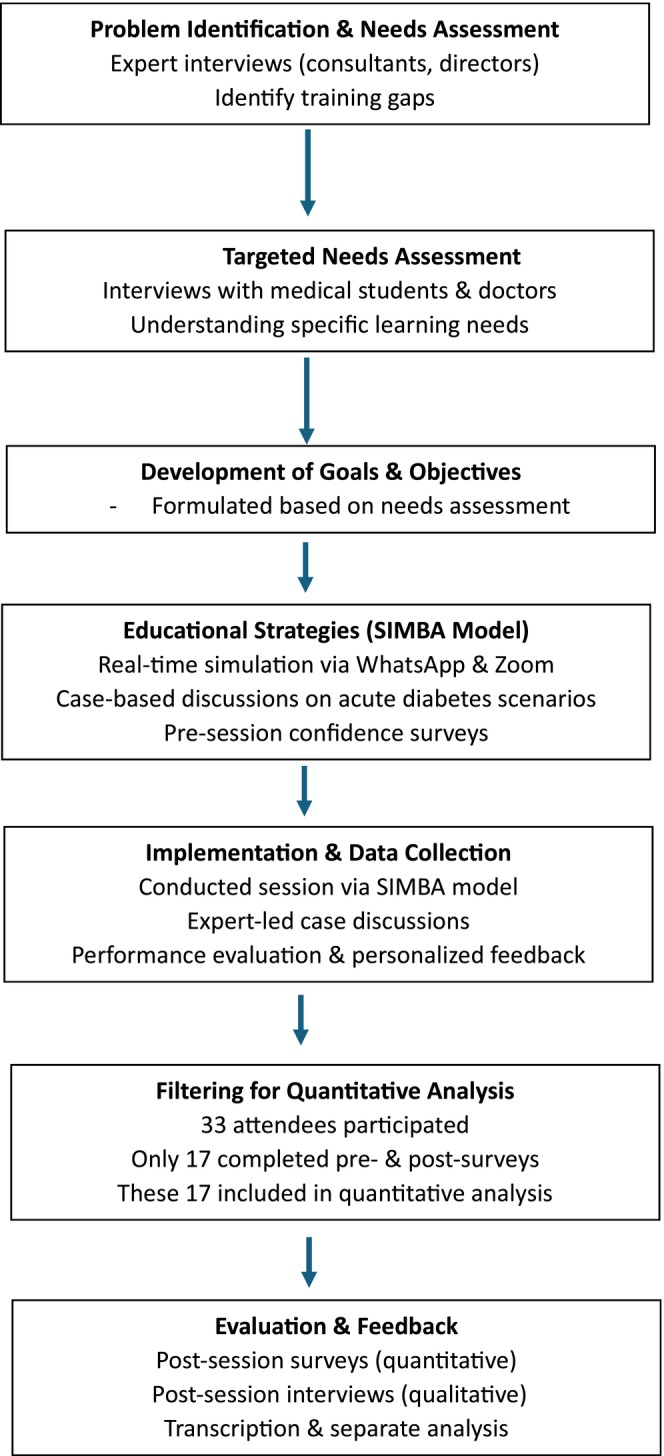
Methodology flow chart for the acute diabetes session.

This study, conducted from July to October 2024, involved a core team comprising medical students, resident doctors and a consultant supported by a larger team of medical students to design and deliver the educational intervention. The Science, Technology, Engineering and Mathematics Ethics Committee at the University of Birmingham granted ethical approval (ERN_2850‐Jul2024). All medical students and resident doctors interested in training in acute diabetes were invited to participate. This was done through flyers, social media promotions and advertisements on various society webpages. We adopted Kern's 6‐Step curriculum development approach to develop and deliver the session^7^ (Figure 1).

### 2.1 Step 1: Problem Identification and General Needs Assessment

We conducted virtual semi‐structured interviews with experts, including consultants and clinical directors, to identify training gaps in acute diabetes management and their perspectives on the role of simulation‐based training in this area. Informed consent was obtained from participants in steps 1 and 2, and the recordings were retained in Microsoft OneDrive, accessible only by the core committee. The interviews lasted, on average, 20 min and were then transcribed verbatim to ensure accuracy.

### 2.2 Step 2: Targeted Needs Assessment

Medical students and resident doctors who registered to attend the conference were invited for an interview to understand their specific needs in managing acute diabetes. Those who expressed interest in participating were interviewed. These interviews were conducted and transcribed as described in Step 1.

### 2.3 Step 3: Development of Goals and Objectives

The objectives of the educational session were formulated based on the findings from the needs assessments.

### 2.4 Step 4: Educational Strategies

The Simulation via Instant Messaging for Bedside Application (SIMBA) model was adopted to deliver the educational intervention. A detailed description of the model has been published previously.^8^ The SIMBA model is a real‐time simulation‐based medical training programme that uses WhatsApp and Zoom to run sessions. It has been peer‐reviewed and proven to be an effective method of information dissemination, providing interactive cases and individualised feedback for participants.

**FIGURE 2:**
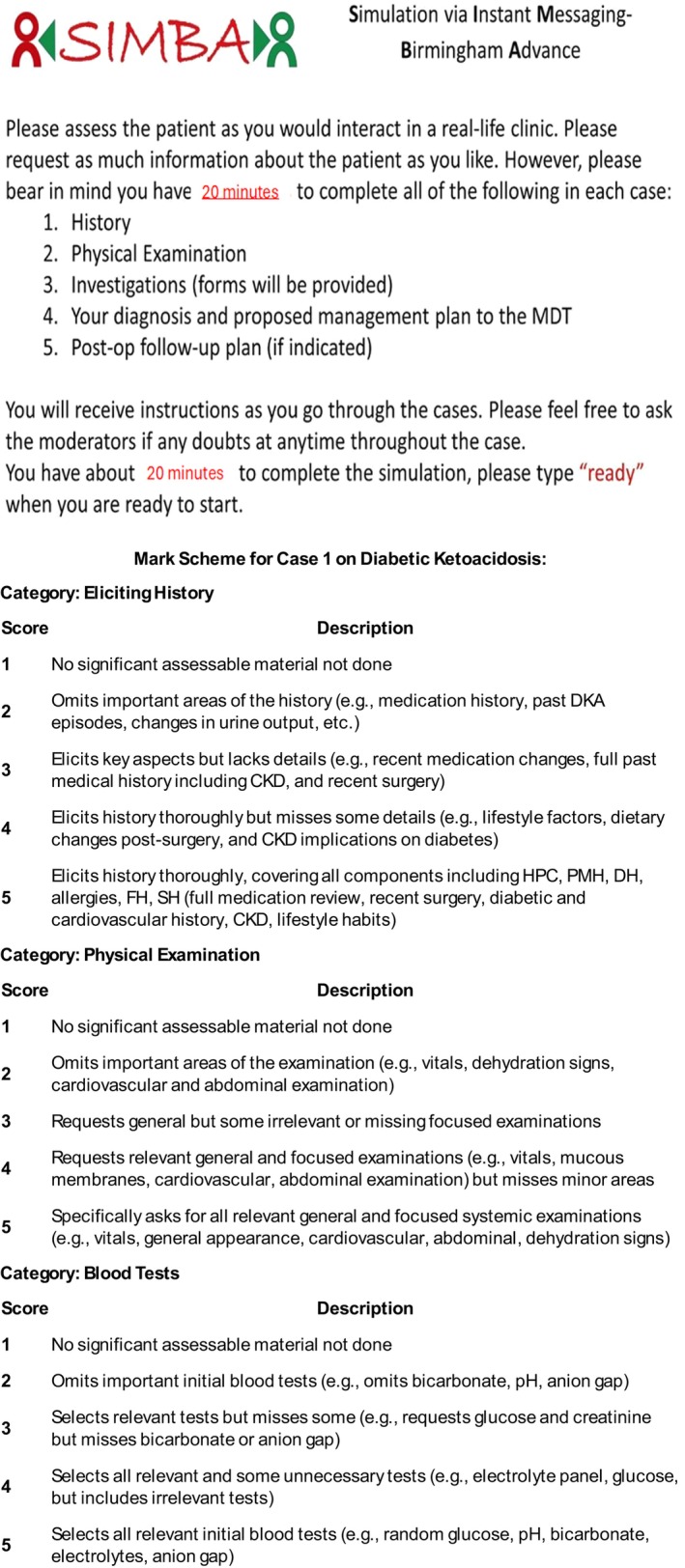
Instructions given to SIMBA participants prior to starting the cases and an example mark scheme used for the simulation on diabetic ketoacidosis.

The session agenda included keynote speakers and case‐based simulations focused on diabetic ketoacidosis (DKA), hyperglycaemic hyperosmolar state (HHS), steroid‐induced hyperglycaemia and other emergencies. Participants were provided with a simulated patient scenario and then asked to interact with moderators virtually via WhatsApp as if they were approaching the clinical case in real life. This included gathering a history, examination findings, ordering and interpreting investigations before arriving at a diagnosis and management plan (Figure 2). The case studies incorporated social determinants of health to provide context for patient care. The session involved simulation‐based problem‐solving through WhatsApp and case discussions facilitated by trained experts in‐person. Participants completed a survey on their confidence for various acute medical scenarios pre‐ and post‐session. After the session, the simulation moderator evaluated participants' performance across various domains using a global rating scale endorsed by the scenario expert. Personalised feedback was then provided to each participant, highlighting areas of strength and opportunities for improvement.

### 2.5 Step 5: Implementation

Author KP managed the conference program using Gantt charts and regular meetings to ensure coordination and workload distribution. AK supervised facilitator training and moderation. AB handled advertising and participant registration. Other administrative leads supported logistics, including form creation and outreach for endorsements. This structured approach facilitated clear communication and operational efficiency, enabling the team to identify facilitators and barriers in delivering the sessions and to design interventions to address the latter.

### 2.6 Step 6: Evaluation and Feedback

After the session, feedback was collected from stakeholders and participants through post‐simulation surveys and interviews. The interviews were conducted and transcribed as described in Step 1.

### 2.7 Analysis

Two study authors independently analysed transcripts from steps 1, 2 and 6 to identify recurring themes. A preliminary codebook was developed after analysing the first transcript from each step and was collaboratively refined to ensure consistency in the coding process. Following this, all transcripts were imported into NVivo 14 and systematically coded for qualitative analysis. The codes were then subjected to thematic analysis, enabling the identification, review and interpretation of patterns and overarching themes. The Kappa coefficient was calculated to assess intercoder agreement.

Quantitative analysis was conducted using Stata (Stata/SE 16.0 for Mac). Participants were scored based on data extracted from pre‐ and post‐simulation surveys, with only those completing both surveys included in the analysis. The findings are reported in frequencies and percentages to the nearest decimal point. Statistical comparisons of pre‐ and post‐simulation confidence levels were performed using the Wilcoxon signed‐rank test, with a 95% confidence interval (*p* < 0.05) considered statistically significant.

## 3 RESULTS

### 3.1 Findings from Problem Identification and General Needs Assessment

Eight stakeholders from diverse medical backgrounds were interviewed, including consultants, clinical directors and national recruitment leads. The codes generated thematically resulted in four domains. A comprehensive summary of these codes and themes is presented in Supplement 2.

#### 3.1.1 Domain 1: Stakeholder Engagement and Multidisciplinary Collaboration in Diabetes Care Education

While there was good recognition and management of acute diabetes presentation at initial admission, the ongoing care within inpatient settings or the identification of similar cases within the inpatients was comparatively suboptimal. Additionally, there was a lack of awareness around guideline updates among resident doctors, which, compounded by frequent rotations, poses challenges in keeping them updated on specific guidelines. The stakeholders advocated routine case‐based discussions around the practical knowledge application and inclusion of trainees from other specialities within multidisciplinary discussions to address the knowledge gaps in acute diabetes. They also highlighted the potential of technological tools—such as artificial intelligence, e‐learning modules, educational videos and phone apps—as valuable resources for supporting future medical education.

#### 3.1.2 Stakeholder Interview 2

‘I think everyone's good at recognising DKA on the front door, so they know what to do. It's when it's on the inpatient side, I guess it's the problem.’

#### 3.1.3 Domain 2: Implementing Core Principles of Acute Diabetes Management in Clinical Practice

In addition to the core knowledge, stakeholders emphasised the importance of seeking help and avoiding assumptions in acute diabetes management. One stakeholder noted that healthcare professionals have polarised attitudes towards diabetes, ranging from fear to indifference.

#### 3.1.4 Stakeholder Interview 3

‘Some there are data to suggest that some people think they do know when in fact they don't know. So the implementation of the guidelines is not as good as it could be, or should be, particularly when people move out of the emergency areas into general wards.’

#### 3.1.5 Domain 3: Optimizing Simulation‐Based Education in Acute Diabetes Management

The interviewees acknowledged the potential of simulation‐based education to provide a safe environment for participants to apply their knowledge, build confidence and reduce future errors.

#### 3.1.6 Stakeholder Interview 2

‘It allows you for mistakes because if you do make a mistake, it's not going to be a disastrous consequence because it's, you know, wasn't real’.

#### 3.1.7 Domain 4: Addressing Barriers to Healthcare Education in Acute Diabetes Management

Stakeholders agreed that while simulations are valuable for healthcare training, their integration into existing programs presents several challenges. Firstly, simulations are resource‐intensive, necessitating substantial funding that requires convincing more stakeholders of their applicability in medical education. Additionally, there is resistance to change, as simulation‐based learning is relatively new and requires greater public acceptance. To address this, stakeholders suggested wider promotion of simulation events. Time constraints due to academic responsibility and clinical duties further hinder participation. To mitigate this, stakeholders suggested embedding simulation‐based learning into regular teaching sessions or making it part of competency qualification.

#### 3.1.8 Stakeholder Interview 1

‘…if you want to run simulation you need SIM facilities. And SIM facilities, I mean we have SIM facilities in [redacted] for example, but I know for a fact that they're heavily oversubscribed and it's a challenge if you want to run something new … within the existing facilities…’

#### 3.1.9 Stakeholder Interview 6

‘I think it's like anything at the one of the things that we will have to do is to advertise this and have a buy in from the organizations. So basically, right from the universities to the clinical teachers and also the healthcare trusts they need to be made aware of the fact that SIMBA training is available and that can be easily accessible and that they should be able to offer it to their this’.

### 3.2 Targeted Needs Assessment: Medical Student and Resident Doctor Interviews

15 interviews were performed, and a comprehensive summary of the codes and domains generated through thematic analysis is presented in Supplement 4.

#### 3.2.1 Domain 1: Education and Training in Acute Diabetes

While most interviewees had received theoretical training on acute diabetes, they highlighted the gap in safe spaces for the practical application of this knowledge. Participants highly valued small group teaching, case‐based discussions and stepwise learning approaches. Simulation‐based learning was frequently mentioned as particularly beneficial in enhancing their understanding and better preparing them for real‐life situations.

#### 3.2.2 Participant 009″

‘…I think more practice of simulation‐type scenarios so that we can better identify what DKA might look like’.

#### 3.2.3 Domain 2: Benefits of Simulation‐Based Learning

Most participants had prior experience with simulation‐based learning, which they described as invaluable for practising and applying theoretical knowledge in a safe, controlled environment. This approach was particularly beneficial in preparing them for acute diabetes presentations. Additionally, targeted and actionable feedback provided after each session was highly valued, allowing participants to reflect and refine their skills effectively before encountering real‐life scenarios.

#### 3.2.4 Participant 004

‘…I knew that it was a safe environment at the end of the day and doing something like that compared to going straight into an emergency scenario with having never done a simulation like that before…’

#### 3.2.5 Participant 009

‘…being giving like really targeted feedback, like specific things that you can change for next time’.

#### 3.2.6 Domain 3: Barriers and Facilitators to Participation in SIMBA‐Related Events

Most participants had previous experience with SIMBA‐related events and shared highly positive feedback about their involvement. While many discovered SIMBA through word‐of‐mouth or university bulletins, there was a consensus on the need for wider advertisement. Participants felt that broader promotion could help more medical students benefit from this valuable opportunity to reinforce their medical education.

#### 3.2.7 Participant 013

‘I feel like sometimes they're not advertised. I feel like it's kind of directed towards junior doctors and that type of thing even though they do say med students. But I think I've heard less med students do it rather than junior doctors and things. So maybe just making it where … it can be a visual resource and see what you know. And that is a safe environment like no one's gonna score you on it’.

#### 3.2.8 Domain 4: Recommendations and Future Directions

Participants shared various motivations for attending the DEKODE/SIMBA Conference, including professional development, exploring simulation‐based learning and gaining further experience in medical education. While SIMBA events have traditionally focused on endocrinology, it was recommended that this approach be expanded to other medical and surgical specialities.

#### 3.2.9 Participant 003

‘…whilst this is used for larger endocrinology. From what I've seen so far, it would be interesting to see if there's a different equivalent for different specialties or areas of medicine…’

### 3.3 Goals and Objectives

Based on the results from the general needs (Step 1) and targeted needs (Step 2) assessments, the session was designed based on the following objectives:

- To develop an applied learning intervention identifying key gaps in acute diabetes training and assess participants' change in confidence in managing these scenarios following the intervention.
- To evaluate participants' satisfaction and the intervention's relevance to clinical practice.

### 3.4 Findings from the session

#### 3.4.1 Participant demographics

33 participants attended the session. 17 participants who attended the session completed both the pre‐simulation and post‐simulation questionnaires and were included in this analysis. 15 (88.2%) participants worked in the UK, whereas 2 (11.8%) worked internationally. Of these, 2 participants were final‐year medical students, and the rest were resident doctors. The baseline knowledge and confidence were similar for all participants, as noted by the pre‐SIMBA survey.

#### 3.4.2 Participant confidence levels

**FIGURE 3:**
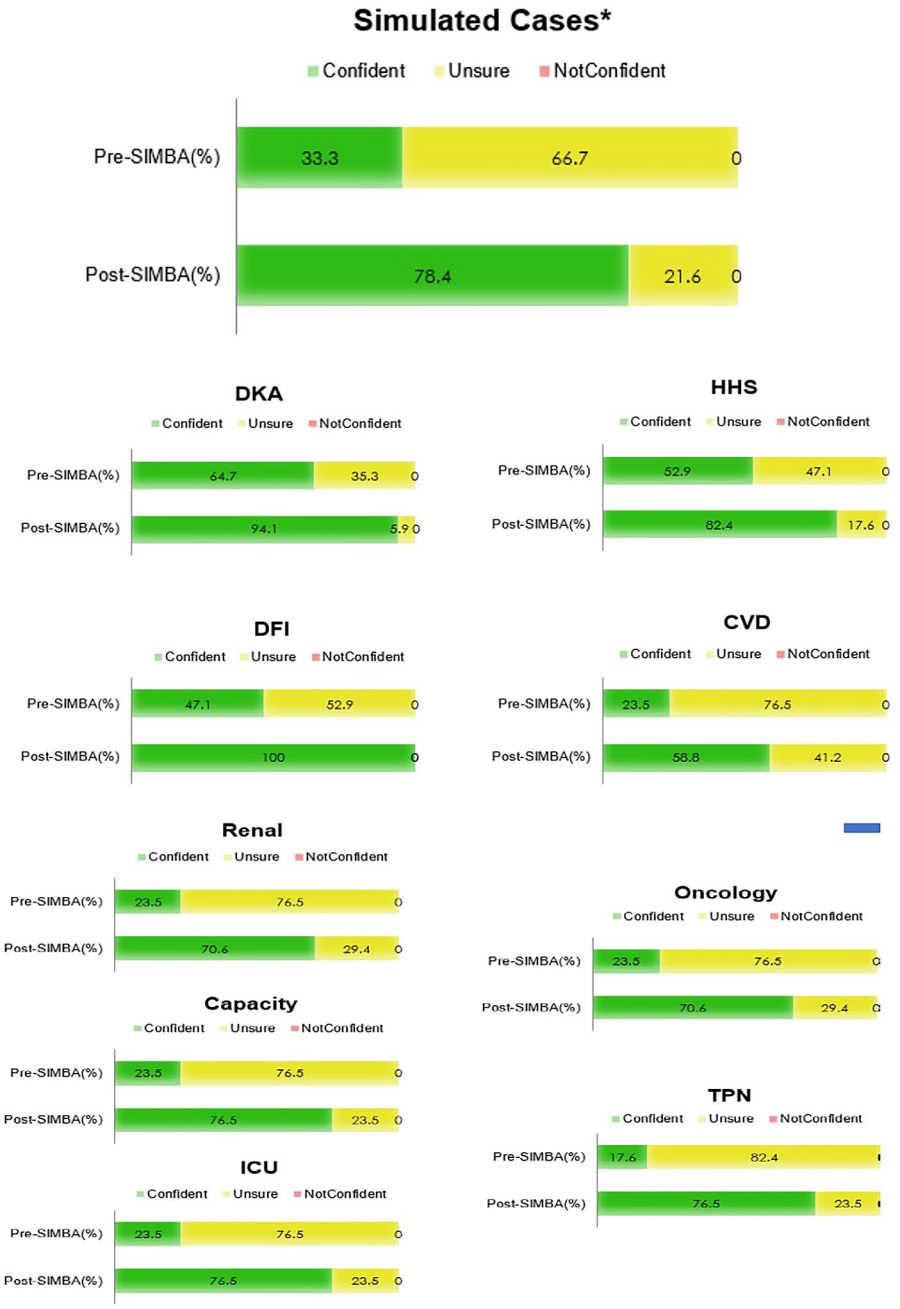
Confidence levels before and after SIMBA simulation case on acute diabetes. Key: DKA, diabetic ketoacidosis; HHS, hyperglycaemic hyperosmolar state; DFI, diabetic foot infection; CVD, cardiovascular disease; ICU, intensive care unit; TPN, total parenteral nutrition.

There was an increase in self‐reported confidence for all acute diabetes scenarios pre vs. post, significance; overall: 33.3% versus 78.4% (*p* < 0.05); diabetic ketoacidosis: 64.7% versus 94.1% (*p* < 0.05); hyperglycaemic hyperosmolar state: 52.0% versus 82.4% (*p* < 0.05); diabetic foot infection: 47.1% versus 100% (*p* < 0.05); cardiovascular disease: 23.5% versus 58.5% (*p* < 0.05); renal disease: 23.6% versus 70.6% (*p* < 0.05); oncology: 23.5% versus 70.6% (*p* < 0.05); capacity: 23.5% versus 76.5% (*p* < 0.05); total parenteral nutrition: 17.6% versus 76.5% (*p* < 0.05); intensive care unit: 23.5% versus 76.5% (*p* < 0.05) (Figure 3).

#### 3.4.3 Feedback and takeaway points

##### Quantitative analysis of feedback

All participants rated the session as excellent or good and the content was impactful at a personal level. 94.1% found the session content impactful at a professional level and of a reasonable length of time for each simulation.

14 (82.3%) participants strongly agreed that the simulated topics were applicable to their clinical practice. All 17 participants found that the simulation improved their knowledge of patient management and practice‐based learning. 16 (94.1%) participants felt their professionalism and patient care had improved, while 15 (88.2%) had improved knowledge of system‐based practice following the simulation.

15 (88.2%) participants found the session engaging 13 (76.5%) agreed that it accommodates their personal learning style. 14 (82.3%) agreed that they would prefer SIMBA to the more traditional lecture‐based learning method. Notably, 16 (94.1%), and participants expressed an intention to attend future similar SIMBA sessions.

##### Global Rating Score (GRS) findings

Participants scored 4.0/5 for history taking, diagnosis and clinical judgement, 3.9/5 for physical examination and investigations, and 3.6/5 for management.

### 3.5 Thematic analysis of post‐session interviews with medical students and resident doctors

Post‐session interviews were conducted between 1 and 2 weeks following the session. From the 15 post‐session interviews with medical students and resident doctors, there were four primary domains. The full thematic analysis and themes derived from the interviews can be found in the table (Supplement 8).

#### 3.5.1 Domain 1: Enhanced Understanding and Confidence in Acute Diabetes Management

Overall, attendees agreed that there was a good representation of areas in acute diabetes management during the session. Both medical students and resident doctors reported an improvement in knowledge of end‐of‐life care, post‐surgery care, diabetic foot disease, steroid‐induced hyperglycaemia, DKA and HHS. Notably, resident doctors expressed an increased confidence in applying the skills or knowledge gained from the simulation in clinical practice. Some additional support or resources suggested by resident doctors included more opportunities to undertake simulation‐based training, as well as resources outlining a ‘stepwise approach’ for managing acute diabetes on a summative document.

Resident doctor 016: ‘So I think I definitely do feel more confident in applying those things that I've learned purely because obviously, if I've simulated it once. It's a little bit like, “see one do and teach one.” I've learned something new, and it helped me retain things that I already knew, but being able to apply them to a scenario like DKA. And I think if I do get presented with some of those rarer cases. For example, the NG feed scenario. I think I'm a lot more confident now to be able to even do start the initial management of it, whereas before I would have perhaps just gone straight to a senior without trying to do something myself. So it's definitely made me more confident’.

#### 3.5.2 Domain 2: Challenges and Limitations of Simulation‐Based Learning

Although there was a wide range of cases in the session, resident doctors identified some areas that were underrepresented during the session, including antenatal and perinatal diabetic care, diabetic remission, hypoglycaemia and perioperative diabetic care. A particular challenge faced by medical students and virtual participants during the work was technical difficulties, culminating in a suboptimal virtual experience, particularly during expert discussions.

Medical student 014: ‘There was some technical issues in the sense that participants and I were unable to hear. Some of the talks that were happening due to audio issues when we joined in and zoom that you know, affected the user experience of the conference’.

#### 3.5.3 Domain 3: Role of Simulation in Developing Clinical and Problem‐Solving Skills

Resident doctors described the session as highly relevant to their stage of training and identified aspects which will be useful for their future clinical practice. Some highlighted aspects included knowledge on advanced care planning and current guidelines, as well as practical aspects such as always remembering to check the feet of a person with diabetes. Attendees felt more confident in using a clear structure when interacting with patients clinically. Specific skills developed by resident doctors included critical thinking skills, selecting relevant investigations and delivering holistic care to patients.

Resident doctor 016: ‘Guidelines will be something we see a lot as an F1, F2 and beyond. So, being able to watch consultants who help compose these guidelines, discuss them together and explain the reasonings for why certain medications weren't approved, etc. Why certain thresholds were needed was very useful for me. Really show me what clinical practice is going to be about with regards to using guidelines and following protocols’.

#### 3.5.4 Domain 4: Feedback and Suggestions for Improvement of Simulation Sessions

Resident doctors found the session very well organised, with simulations being well‐timed and reflective of real‐life scenarios. Notably, expert discussions were identified as a highlight of the session. However, they felt there was a lack of teamwork during the simulation, and some suggested having one electronic device per table for in‐person attendees, encouraging participants to work together on each case.

Resident doctor 016: ‘So I think there's a couple of aspects to that. I don't know whether this simulation could be altered in order to have, instead of one‐to‐one moderator working through a scenario, whether it could be simulated within a group. Obviously, it's all resource dependent whether you can have, like one laptop per table and people working together, discussing what you would happen. And then having to making a group discussion and group decision about the response to the moderator. I think that would help with teamwork’.

### 3.6 Thematic analysis of post‐session interviews with experts who delivered the session

Four primary domains emerged from the post‐session interviews with stakeholders. The table (Supplement 9) provides the full thematic analysis and themes derived from these interviews.

#### 3.6.1 Domain 1: Benefits and Challenges of Simulation‐Based Learning

The experts identified several benefits of simulation‐based learning for acute diabetes care, describing it as an environment reflective of real‐life scenarios that allow mistakes to be made. Notably, experts found SIMBA to be an effective method of learning for acute diabetes care, enabling trainees' confidence and familiarity with acute diabetes management to increase. Some foreseen barriers to scaling simulation‐based learning in acute diabetes care include resident doctors' demanding schedules and the extensive range of topics trainees must cover, as well as funding, suitable locations and staffing resources.

Expert 011: ‘[regarding SIMBA] I think it's a great platform is (A) cost effective (B) inclusive (C) iterative and (D) impactful’.

#### 3.6.2 Domain 2: Improving Simulation Engagement and Realism

Experts found that the management principles and decision‐making aspects of the simulated cases were well aligned with real‐life acute diabetes scenarios.

There were, however, suggestions on how to improve future sessions on diabetes: For participants, the simulations could be more targeted and tailored to either a medical student, resident doctor or registrar level. For stakeholders, the experience could be improved by increasing the length of discussion and interaction for each case. Additionally, resources that could be useful in acute diabetes care training include simplified flow charts summarising management, as well as QR codes or apps to enable quick access to guidelines.

Expert 008: ‘It was extremely well organised. It was extremely well attended. And the topics were all very relevant. And most importantly, it was engaging with those who were involved’.

#### 3.6.3 Domain 3: Facilitating Adoption and integration of SIMBA in Training

Stakeholders were in favour of the integration of SIMBA into routine training for acute diabetes, suggesting methods on how to achieve this. SIMBA could be incorporated into both the formal induction training programme for resident doctors and the formal registrar training programme, providing a dedicated, regular training opportunity within the curriculum. Factors that could facilitate this integration include increasing awareness of SIMBA sessions through ‘word‐of‐mouth’, national forums and professional platforms such as ABCD, YDEF and LinkedIn.

Expert 009: ‘It fits very well. You absolutely need to take this. And there is competition in this space, you know. So what you and others need to do is to take this into a national forum. I keep going back to ABCD simply because I can't think of anyone else. But you could put this under the ABCD umbrella for diabetes. I know you do lots of other non‐diabetes teaching. but for diabetes, if you put it on the ABCD, you would get much more engagement’.

#### 3.6.4 Domain 4: Networking and Professional Collaboration

The experts who participated in the session found it to be an excellent opportunity for networking with colleagues, helping to both strengthen existing professional relationships and establish new ones. To improve networking and collaboration further, stakeholders suggested that more socialisations should be encouraged between consultants and resident doctors, as well as between consultants and medical students.

Expert 009: ‘Among participants, so I think, encouraging them, especially in the break time and lunchtime, to speak to the different consultants. Because the facilitators probably go through their case in a bit more detail. But talking to experts and probably discussing things like the gaps in their learning or things that they haven't quite understood, or finding out what else is available in terms of training days or things that they could attend. So I think it's quite useful to use those opportunities’.

## 4 DISCUSSION

This study demonstrated that a targeted, simulation‐based program using the SIMBA model significantly improved healthcare professionals' confidence and competence in managing acute diabetes scenarios beyond the initial emergency phase.

Simulation‐based learning has been increasingly recognised in medical education literature as an effective tool for enhancing knowledge retention, clinical decision‐making and confidence in real‐world applications.^9^ The SIMBA model, leveraging both online and in‐person elements, addresses concerns noted in recent literature regarding the challenges of creating ‘realistic’ virtual simulations.^10^ By offering hybrid formats and in‐depth debriefing with senior clinicians, this study's approach overcame barriers of limited realism often cited in virtual‐only simulation models, thereby enhancing participant engagement and retention.^11^

Previous SIMBA sessions predominantly focused on the initial acute management phase.^12^ Focusing on sustained management, this study contributes a new perspective, aligning with calls in the literature for more complex, longitudinal simulation models. This approach supports the holistic development of healthcare professionals' skills, aligning with growing recommendations in healthcare training to focus on continuity of care and patient‐centred practices, which are crucial in managing chronic, complex conditions like diabetes.^13^

While we involved nine experts in this session, the SIMBA sessions can be run by just one expert with similar gains in confidence as demonstrated in our previous sessions.^14^ The study's strengths lie in its rigorous design and adaptability, particularly Kern's 6‐Step Approach,^15^ which structured each phase of curriculum development based on needs assessment and evidence‐based educational strategies.^16^ Another significant strength was the inclusion of social determinants of health within the simulation cases, offering participants context that often impacts diabetes management.^17^

Technical audiovisual issues impacted some participants' experience. This issue, common in hybrid simulations, may limit engagement quality and introduce variability in learning outcomes between in‐person and online participants.^17^ Future iterations could mitigate this by investing in more robust technical infrastructure to enhance the virtual component's quality.

As this is the first simulation session of its kind in diabetes, the findings may not yet be generalisable. However, previous work with SIMBA has demonstrated reproducibility^18^ and sustainability of outcomes,^19^ and we anticipate that SIMBA diabetes may yield similar benefits. Nonetheless, further research is required to evaluate the effectiveness and scalability of this approach across multiple sessions and diverse participant groups. This could be achieved through future iterations of the model and its application in a broader range of settings.

Furthermore, we did not compare SIMBA with traditional teaching methods such as lectures or seminar groups. Also, while the study assessed immediate confidence and knowledge gains, future studies are planned to consider longitudinal assessments to evaluate whether the skills gained in simulation translate into improved confidence and, thereby, patient care over time and to explore if the training model impacts system‐level metrics such as discharge efficiency and readmission rates.

## 5 CONCLUSION

This study presents a model for addressing gaps in acute diabetes education. Stakeholders highlighted the need for training beyond initial care, focusing on guideline implementation and long‐term management. SIMBA's simulation‐based program improved participant confidence and understanding, with 94.1% affirming its relevance. The program aligns with healthcare priorities by emphasizing patient‐centred, integrated care.

## AUTHOR CONTRIBUTIONS

KP led project management and team coordination, supervised analysis, created the initial draft and finalised the manuscript. AK and AB performed qualitative data analysis, created codebooks, piloted data coding and ensured the inter‐reliability of qualitative results. ALJY, NP and AS focused on ensuring smooth program delivery and contributed to the initial draft. AS performed quantitative data analysis and created visual representations of the quantitative data. SS provided support for qualitative analysis and contributed to creating the initial draft. PK is the senior author and supervisor, provided overall project supervision, validated the study design and oversaw all aspects of the research. All members of the DEVI collaboration contributed equally to the design and delivery of the simulation, manuscript review and editing. The members of this collaboration thus warrant authorship on this study, and this includes Rahul Sagu, Josh Banerjee, Maiar Elhariry, Anu Ann Abraham, Sulmaaz Qamar, Ketan Dhatariya, Gerry Rayman, Mujahid Saeed, Srikanth Bellary, Jason Cheung, Rajeev Raghavan, Feaz Bawbah and Mohammed Ali Karamat. All authors participated in the investigation by conducting interviews and distributing surveys, contributing to methodology design, reviewing transcripts and reviewing the final manuscript. All authors have read and approved the final version of the manuscript.

## FUNDING INFORMATION

The ABCD Sanofi DKA Collaborative Project Grant awarded by the Association of British Clinical Diabetologists and the educational meeting grant from the Health Education West Midlands awarded to PK supported this study. PK also receives support from the National Institute for Health and Care Research (NIHR) through his Advanced Clinician Scientist Fellowship (NIHR303671). Additionally, he is supported by the Midlands Patient Safety Research Collaboration (PSRC) and the Race, Equity, and Diversity in Careers Incubator. The views expressed in this study are those of the author and do not necessarily reflect the official positions of the NIHR or the Department of Health and Social Care.

## CONFLICT OF INTEREST STATEMENT

The authors declare no conflicts of interest.

## Supporting information


Data S1:

Data S2:

Data S3:

Data S4:

Data S5:

Data S6:

Data S7:

Data S8:

Data S9:

## Data Availability

The data that support the findings of this study are available from the corresponding author upon reasonable request.

## References

[1] Dhatariya K , Mustafa OG , Rayman G . Safe care for people with diabetes in hospital. Clin Med. 2020;20(1):21‐27. 10.7861/clinmed.2019-0255 PMC6964176 31941727

[2] Galindo RJ , Trujillo JM , Low Wang CC , McCoy RG . Advances in the management of type 2 diabetes in adults. BMJ Med. 2023;2(1):e000372. 10.1136/bmjmed-2022-000372 PMC10481754 37680340

[3] Umpierrez GE , Davis GM , ElSayed NA , et al. Hyperglycaemic crises in adults with diabetes: a consensus report. Diabetologia. 2024;67(8):1455‐1479. 38907161 10.1007/s00125-024-06183-8 PMC11343900

[4] Kangas S , Jaatinen P , Metso S , Paavilainen E , Rintala TM . Students' perceptions of interprofessional collaboration on the care of diabetes: a qualitative study. Nurse Educ Pract. 2021;53:103023. 33894489 10.1016/j.nepr.2021.103023

[5] Lateef F . Simulation‐based learning: just like the real thing. J Emerg Trauma Shock. 2010;3(4):348‐352. 21063557 10.4103/0974-2700.70743 PMC2966567

[6] Jee M , Murphy E , Umana E , et al. Exploring barriers and enablers to simulation‐based training in emergency departments: an international qualitative study (BEST‐ED study). BMJ Open. 2023;13(9):e073099. 10.1136/bmjopen-2023-073099 PMC10481749 37669835

[7] Thomas P . Curriculum Development for Medical Education. Johns Hopkins University Press; 2022.

[8] Davitadze M , Ooi E , Ng CY , et al. SIMBA: using Kolb's learning theory in simulation‐based learning to improve participants' confidence. BMC Med Educ. 2022;22(1):116. 35193557 10.1186/s12909-022-03176-2 PMC8861259

[9] Dhawan S . Online learning: a panacea in the time of COVID‐19 crisis. J Educ Technol Syst. 2020;49(1):5‐22.

[10] Pascucci RC , Weinstock PH , O'Connor BE , Fancy KM , Meyer EC . Integrating actors into a simulation program. Simul Health. 2014;9(2):120‐126. 10.1097/SIH.0b013e3182a3ded7 24096918

[11] Bulte C , Betts A , Garner K , Durning S . Student teaching: views of student near‐peer teachers and learners. Med Teach. 2007;29(6):583‐590. 17922356 10.1080/01421590701583824

[12] D'Souza MS , Labrague LJ , Karkada SN , Parahoo K , Venkatesaperumal R . Testing a diabetes keotacidosis simulation in critical care nursing: a randomized control trial. Clin Epidemiol Glob Health. 2020;8(4):998‐1005.

[13] Barnett K , Mercer SW , Norbury M , Watt G , Wyke S , Guthrie B . Epidemiology of multimorbidity and implications for health care, research, and medical education: a cross‐sectional study. Lancet. 2012;380(9836):37‐43. 22579043 10.1016/S0140-6736(12)60240-2

[14] Morgan G , Melson E , Davitadze M , et al. Utility of simulation via instant messaging – Birmingham advance (Simba) in medical education during Covid‐19 pandemic. J Royal Coll Physic Edinburgh. 2021;51(2):168‐172. 10.4997/JRCPE.2021.218 34131679

[15] Sweet L , Palazzi D . Application of Kern's six‐step approach to curriculum development by global health residents. Educ Health. 2015;28(2):138‐141. 10.4103/1357-6283.170124 26609014

[16] Levy NK , Park A , Solis D , et al. Social determinants of health and diabetes‐related distress in patients with insulin‐dependent type 2 diabetes: cross‐sectional, mixed methods approach. JMIR Form Res. 2022;6(10):e40164. 36222807 10.2196/40164 PMC9607916

[17] Eija N , Sari PS , Kristina M , Tiina T , Jonna J , Heli‐Maria K . The experiences of health sciences students with hybrid learning in health sciences education—a qualitative study. Nurse Educ Today. 2024;132:106017. 37948975 10.1016/j.nedt.2023.106017

[18] Wallett L , Chen W , Thomas L , et al. Developing a simulation‐based learning model for acute medical education during COVID‐19 pandemic with simulation via instant messaging – Birmingham advance (SIMBA). BMJ Open Qual. 2022;11(2):e001565. 10.1136/bmjoq-2021-001565 PMC8995572 35396253

[19] Zhou D , Davitadze M , Ooi E , et al. Sustained clinical knowledge improvements from simulation experiences with simulation via instant messaging—Birmingham advance. Postgrad Med J. 2023;99(1167):25‐31. 36947426 10.1093/postmj/qgac008

