# Supplementary material for: Simulation‐based training significantly improved confidence and clinical skills of resident doctors in acute diabetes management
Source: Diabet Med. 2025 Jun 17;42(9):e70068. doi: 10.1111/dme.70068 (PMC12352711; doi:10.1111/dme.70068)
Supplement: Supplementary file 1 — Data S1: [file DME-42-e70068-s001.docx]

**SUPPLEMENT 1: Interview Questionnaire for SIMBA Acute Diabetes: General Needs Assessment**

**Section 1: Introduction (5 mins)**

1. Introduce the interviewer/facilitator

2. Thank the participant for participating

3. Check participant’s audio and video are working

4. Confirm they are happy for us to record

START RECORDING

1. Reintroduce the interviewer/facilitator - "Hi, I am _____, one of the facilitators for this research."

2. "Thank you for participating. Do you consent to the audio recording of your interview and using your pseudonymized quotes in research reports and publications?"

3. "This interview aims to understand your experiences and expectations from simulation based learning for acute diabetes. There are no right or wrong answers. Your experiences and opinions are important and valid, and that is why we have invited you to speak with us today."

4. "I have some questions I will ask to lead the conversation; otherwise, we want you to do the talking. If you think something is important, please bring it up, and if you don’t think a question or topic is relevant, please say so."

5. "You can ask me to pause the recording at any time, and you are also free to leave the interview at any time.

6. "If you want to find out more about how we handle your data, please refer to the participant information sheet that was emailed to you beforehand. If you have any questions about how we handle your data, please do ask me or get in contact with me via email."

**Section 2: Background and Motivation**

1. Tell me a bit about yourself. Where do you currently work and at what level?
2. How are you involved in educating healthcare professionals and/or people with diabetes about acute diabetes?

**Section 3: Knowledge and Experience**

1. What aspects of acute diabetes management do you think are done well in current practice?
2. What key principles of acute diabetes management do you think healthcare professionals should be aware of?
3. Do you have any suggestions or feedback on how medical schools or training programmes can better address the needs and knowledge gaps of students in the area of acute diabetes?
4. What support or resources do you feel would be beneficial for medical students and junior doctors to enhance their understanding and skills in acute diabetes management?

**Section 4: Experiences with Simulation-Based Learning**

1. Have you participated in any simulation-based learning during your medical education or within the NHS? If so, can you describe your role and the outcomes?
2. What aspects of these simulation-based learning experiences were done well?
3. What do you think could be improved in simulation-based learning?

**Section 5: Awareness and Participation in SIMBA**

1. Have you heard about SIMBA before?
2. Where did you hear about it?
3. Have you attended a SIMBA session?
   1. If yes, proceed to Section 6
   2. If no, proceed to Section 7

**Section 6: SIMBA Attendance**

1. What was your experience with SIMBA?
2. What aspects of SIMBA were done well?
3. What do you think could be improved in SIMBA?

- Go to Section 8

**Section 7: Information about SIMBA**

1. Provide information about SIMBA (share YouTube links if needed).
2. What are your thoughts about SIMBA for medical training for healthcare professionals?

**Section 8: Scalability and sustainability**

1. What areas of acute diabetes could be taught using simulation?
2. What factors do you think facilitate attending these sessions?
3. What do you see as the barriers to attending these sessions?
4. How can we embed simulation of acute diabetes scenarios within healthcare training programmes to ensure sustainability?

THANK AND FINISH RECORDING
