# Supplementary material for: Simulation‐based training significantly improved confidence and clinical skills of resident doctors in acute diabetes management
Source: Diabet Med. 2025 Jun 17;42(9):e70068. doi: 10.1111/dme.70068 (PMC12352711; doi:10.1111/dme.70068)
Supplement: Supplementary file 2 — Data S2: [file DME-42-e70068-s008.docx]

**Supplement 2: General Needs Assessment Qualitative Analysis**

Kappa-Coefficient – 0.62

**n- this refers to the number of times the domain was coded in the qualitative analysis.**

*Themes and domains developed from thematic analysis:*

| Codes | Themes | Domains |
| --- | --- | --- |
| Clinical Academics | Roles of Stakeholders | Stakeholder Engagement and Multidisciplinary Collaboration in Diabetes Care Education (n=313) |
| Clinical Director |  |  |
| Consultants |  |  |
| GIRFT Co-Lead |  |  |
| NaDIA Developer |  |  |
| National Recruitment Lead |  |  |
| NHS Diabetes Clinical Lead |  |  |
| Service Lead |  |  |
| Training Program Director |  |  |
| University Horonary Chair |  |  |
| Community education | Educational Methods on acute diabetes |  |
| Guideline updates |  |  |
| Involvement of specialties other than endocrinology |  |  |
| MDT discussions |  |  |
| Simulations |  |  |
| Teaching sessions |  |  |
| Ward-based teaching |  |  |
| Good recognition and management in ED | Positive aspects of current acute diabetes management |  |
| Inpatient management |  |  |
| Patient empowerment |  |  |
| Perioperative diabetic care |  |  |
| Standardised documents |  |  |
| Appropriate monitoring and replacement | Challenges in acute diabetes management |  |
| Awareness spread to other specialties |  |  |
| Diabetic foot |  |  |
| Differentiation between acute hyperglycaemia scenarios |  |  |
| Fluid prescribing |  |  |
| Guideline adherence and implementation |  |  |
| Guideline changes |  |  |
| Handover process |  |  |
| **Hospital acquired days (length of stay)** |  |  |
| Hospital acquired delays |  |  |
| Inappropriate patient support |  |  |
| Poor knowledge around insulin therapy |  |  |
| Poor recognition and management in inpatient.outpatient settings |  |  |
| Rapid turnover of trainee doctors |  |  |
| Understanding around RF that trigger hyperglycaemia |  |  |
| Active teaching | Suggestions to address needs and knowledge gaps in acute diabetes |  |
| Appropriate time allocation |  |  |
| Case-based teaching |  |  |
| Clear curriculum |  |  |
| Guideline familiarization |  |  |
| Inclusion in specialty rounds |  |  |
| Introduction to technology in diabetes |  |  |
| Opportunity-seeking behaviour |  |  |
| Pointers |  |  |
| Practical experience |  |  |
| Practical knowledge application |  |  |
| Routine competency assessment |  |  |
| Simplify complex information |  |  |
| Simulation training |  |  |
| Time with diabetes nurses |  |  |
| Understand pathophysiology |  |  |
| Up-to-date content |  |  |
| Ward-based teaching |  |  |
| Artificial intelligence | Beneficial educational support and resources |  |
| Clinical scenarios |  |  |
| E-learning modules |  |  |
| Educational videos |  |  |
| Evolving roles |  |  |
| Game-based learning |  |  |
| Guideline standardization |  |  |
| Interactive pathways |  |  |
| Phone apps |  |  |
| Teaching materials |  |  |
| Understanding pathophysiology |  |  |
| Ask for help | Key principles of acute diabetes that healthcare professionals should be aware of | Implementing Core Principles of Acute Diabetes Management in Clinical Practice (n=52) |
| Avoid assumptions that one’s know everything |  |  |
| Glucose and ketone monitoring |  |  |
| Glucose threshold |  |  |
| Glucose-lowering medications |  |  |
| HbA1C interpretation |  |  |
| History taking and quick examination |  |  |
| Hypoglycaemia management |  |  |
| Insulin prescription |  |  |
| Never turn your back |  |  |
| Patient support |  |  |
| Prevention measures |  |  |
| Smooth handover process |  |  |
| Recognising acute emergencies and escalating |  |  |
| Fear | Feelings.ideas towards diabetes |  |
| Indifferent |  |  |
| Provide confidence | Importance of educating HCPs on acute diabetes management |  |
| Build confidence | Benefits of simulation training | Optimizing Simulation-Based Education in Acute Diabetes Management (n=184) |
| Easily accessible |  |  |
| Interactive |  |  |
| Practical experience |  |  |
| Practice working under time pressure |  |  |
| Realistic scenarios with various possibilities exposed |  |  |
| Safe environment for trial and error |  |  |
| Technological integrations |  |  |
| No | Previous experience with simulation-based learning |  |
| Yes |  |  |
| Publications | Sources of SIMBA |  |
| Word of mouth |  |  |
| Attract interest | Benefits of SIMBA |  |
| Concise information delivery |  |  |
| Conducive learning environment |  |  |
| Deconstructing complexity |  |  |
| Engagement |  |  |
| Evidence-based |  |  |
| Inclusion of medical students |  |  |
| Multimodal teaching |  |  |
| Self-independent learning |  |  |
| Trigger discussions |  |  |
| Understand and address needs | Thoughts around case preparation |  |
| Diabetes technology | Areas of acute diabetes for simulation-based teaching |  |
| Diabetic foot |  |  |
| DKA |  |  |
| Enteral feeding |  |  |
| HHS |  |  |
| Insulin and its technology |  |  |
| Mental capacity |  |  |
| Monitoring regime |  |  |
| Pre-surgical preparation |  |  |
| Procedural skills |  |  |
| Severe hypoglycaemia |  |  |
| Steroid-induced hyperglycaemia |  |  |
| Building stakeholder buy-in | Limitation for simulation in healthcare training programme | Addressing Barriers to Healthcare Education in Acute Diabetes Management (n=144) |
| Inertia to change |  |  |
| Resource intense |  |  |
| Resources intense |  |  |
| Time and space |  |  |
| A threat of examination | Methods for simulation to be included in healthcare training programme |  |
| Admittance on poor management |  |  |
| Advertisements |  |  |
| Competency qualification |  |  |
| Finance reimbursement |  |  |
| Recorded |  |  |
| Sharing experience |  |  |
| Word of mouth |  |  |
| Brain space | Barriers for attendance |  |
| Caught up with academic confines |  |  |
| Clinical distractions |  |  |
| False learning concepts |  |  |
| Funding |  |  |
| Interests |  |  |
| Time away.pressure |  |  |
| Advertisements | Facilitations for attendance |  |
| Dedicated time slot |  |  |
| Interest |  |  |
| Self-realisation |  |  |
| Understanding agenda |  |  |
| Advertisement | Methods to overcome barriers for attendance |  |
| Incorporation into regular teaching sessions |  |  |
| Constant updating mechanism | Challenges of SIMBA |  |
| Funding |  |  |
| Feedback collection | Recommendations for SIMBA |  |
| Forum for discussion |  |  |
| Human factors involvement |  |  |
| Regular updates |  |  |
| Targeted audience |  |  |
| Visual effects incorporation |  |  |
