# Supplementary material for: Simulation‐based training significantly improved confidence and clinical skills of resident doctors in acute diabetes management
Source: Diabet Med. 2025 Jun 17;42(9):e70068. doi: 10.1111/dme.70068 (PMC12352711; doi:10.1111/dme.70068)
Supplement: Supplementary file 3 — Data S3: [file DME-42-e70068-s003.docx]

**Supplement 3: Interview Questionnaire for SIMBA Acute Diabetes: Targeted Needs Assessment**

**Section 1: Introduction (5 mins)**

1. Introduce the interviewer/facilitator

2. Thank the participant for participating

3. Check participant’s audio and video are working

4. Confirm they are happy for us to record

START RECORDING

1. Reintroduce the interviewer/facilitator - "Hi, I am _____, one of the facilitators for this research."

2. "Thank you for participating. Do you consent to the audio recording of your interview and using your pseudonymized quotes in research reports and publications?"

3. "This interview aims to understand your experiences and expectations from simulation based learning for acute diabetes. There are no right or wrong answers. Your experiences and opinions are important and valid, and that is why we have invited you to speak with us today."

4. "I have some questions I will ask to lead the conversation; otherwise, we want you to do the talking. If you think something is important, please bring it up, and if you don’t think a question or topic is relevant, please say so."

5. "You can ask me to pause the recording at any time, and you are also free to leave the interview at any time.

6. "If you want to find out more about how we handle your data, please refer to the participant information sheet that was emailed to you beforehand. If you have any questions about how we handle your data, please do ask me or get in contact with me via email."

**Section 2: Background and Motivation**

1. Tell me a bit about yourself- where did you do your medical school training and where do you currently work?
2. Why are you interested in attending this SIMBA simulation session on acute diabetes?

**Section 3: Knowledge and Experience**

1. What key principles of acute diabetes management are you familiar with?
2. Please tell me which acute diabetes scenarios have you come across?
3. What are your experiences with managing these scenarios?
4. What education or training have you received to manage acute diabetes scenarios?
5. What was done well?
6. What do you think could be done better?
7. What support or resources do you feel would be beneficial for medical students/ junior doctors like yourself to enhance your understanding and skills in acute diabetes management?
8. Do you have any suggestions or feedback on how medical schools can better address students' needs and knowledge gaps in this area?

**Section 4: Experiences with Simulation Based Learning**

1. Have you participated in any simulation-based learning during your medical education or within the NHS? If so, can you describe your role and the outcomes?
2. What was done well?
3. What do you think could be done better?

**Section 5: Awareness and Participation in SIMBA**

1. Have you heard about SIMBA before?
2. Where did you hear about it?
3. Have you attended a SIMBA session?

    - If yes, proceed to Section 6

    - If no, proceed to Section 7

**Section 6: SIMBA Attendance**

1. What was your experience with SIMBA?

2. What was done well?

3. What could be done better?

Go to Section 8

**Section 7: Information about SIMBA**

1. Provide information about SIMBA (share YouTube links if needed).

SIMBA is a real-time simulation based medical training programme, which uses whatsapp and zoom to run sessions. It has been peer-reviewed and proven to be an effective method of information dissemination, providing interactive cases and individualised feedback for participants.

Youtube link: <https://www.youtube.com/@simbasimulation8047>

2. What are your thoughts about SIMBA for medical training for medical students and junior doctors?

3. What factors facilitate attending these sessions?

4. What are the barriers to attending these sessions?

**Section 8: Skills and Competencies**

1. What do you want to achieve by participating in this SIMBA simulation session?
2. What areas of acute diabetes are you interested in learning about?
3. How do you plan to apply the knowledge and skills gained from this session in your future medical practice?

THANK AND FINISH RECORDING
