# Supplementary material for: Simulation‐based training significantly improved confidence and clinical skills of resident doctors in acute diabetes management
Source: Diabet Med. 2025 Jun 17;42(9):e70068. doi: 10.1111/dme.70068 (PMC12352711; doi:10.1111/dme.70068)
Supplement: Supplementary file 4 — Data S4: [file DME-42-e70068-s002.docx]

**Supplement 4: Targeted Needs Assessment Qualitative Analysis**

Targeted Needs Assessment (no child code) – 0.63

Targeted Needs Assessment (with child codes) – 0.52

*Themes and domains developed from thematic analysis:*

| Code | Theme | Domain (n) |
| --- | --- | --- |
| Medical student | Stage of medical training | Education and Training in Acute Diabetes (337) |
| Resident doctor |  |  |
|  | Areas of interest in acute diabetes |  |
|  | Education or training received around acute diabetes |  |
| Prevention is better than treatment | Key principles of acute diabetes management identified before the conference |  |
| Source for inspiration in getting involved with medical education | Knowledge and skill application post-event |  |
|  | Personal experience around DKA or HHS management |  |
|  | Personal experience around hypoglycaemic management |  |
|  | No previous encounters or experience with acute diabetes scenarios |  |
| Case-based learning | Positive aspects of education or training received around acute diabetes |  |
| Stepwise approach |  |  |
| Diabetic foot disease | Previous encounter with acute diabetes other than DKA and HHS |  |
| Diabetic retinopathy |  |  |
|  | Previous encounter with DKA |  |
|  | Previous encounter with HHS |  |
|  | Previous encounter with hypoglycaemia |  |
| Lack of awareness around HHS | Suggestions or feedback for medical schools to address students' needs and knowledge gaps |  |
| Small group teaching |  |  |
| Infographics | Useful support or resources for medical students to enhance understanding and skills around acute diabetes |  |
| Simulation-based learning |  |  |
| Increased interaction | Recommendations on improving the education or training received around acute diabetes |  |
| Simulation-based learning |  |  |
| Technological advancements |  |  |
|  | Roles in simulation-based learning | Benefits of Simulation-Based Learning (98) |
| Health beliefs | Benefits of simulation-based learning |  |
| Safe environment for learning |  |  |
| Safe environment | Outcomes in simulation-based learning |  |
| Useful feedback |  |  |
| Discussion with experts | Positive aspects about SIMBA events |  |
| Step-by-step guidance |  |  |
|  | Previous experience with simulation-based learning |  |
| Lack of advertisement | Barriers to attending SIMBA-related events | Barriers and Facilitators to Participation in SIMBA-related Events (85) |
| Technological issues |  |  |
| Confidential space | Factors that facilitate SIMBA attendance |  |
| Virtual platform to enable good time management |  |  |
| Word of mouth |  |  |
|  | Previous attendance to SIMBA events |  |
|  | Previous experience with SIMBA |  |
|  | Sources that introduce SIMBA |  |
| Communication | Goals for attending the conference | Recommendations and Future Directions (107) |
| CV development |  |  |
| Gain further insight on simulation-based learning |  |  |
| Gain more experience as a moderator |  |  |
| Gain more experience in medical education |  |  |
| Medical education | Reasons for attending the conference |  |
| Passion for endocrinology |  |  |
| Post-workshop discussions |  |  |
| Research opportunities |  |  |
| Greater breadth | Recommendations for future SIMBA events |  |
| Structural guidance on the approach |  |  |
|  | Recommendations for future simulation-based learning |  |
