# Supplementary material for: Simulation‐based training significantly improved confidence and clinical skills of resident doctors in acute diabetes management
Source: Diabet Med. 2025 Jun 17;42(9):e70068. doi: 10.1111/dme.70068 (PMC12352711; doi:10.1111/dme.70068)
Supplement: Supplementary file 5 — Data S5: [file DME-42-e70068-s005.docx]

**Supplement 5: Agenda for DEKODE/SIMBA Acute Diabetes Conference and Case Transcripts**

| Time | Topic | Speaker | Chair |
| --- | --- | --- | --- |
| 08:30 – 09:00 | Registration- Angelica Sharma | | |
| 09:00 - 09:05 | Introduction and Welcome | Kalyaani Persad, University of Birmingham, UK | [Punith Kempegowda, University Hospitals Birmingham, UK](https://www.birmingham.ac.uk/staff/profiles/applied-health/kempegowda-punith) |
| 09:05 – 09:30 | Basics of Diabetes Management in the Inpatient setting | [Ketan Dhatariya,Consultant in Diabetes Norfolk & Norwich Hospitals, UK](https://profketandhatariya.com/) |  |
| 09:30 - 09:40 | Explaining SIMBA model and pre-SIMBA survey | Rahul Sagu, University of Birmingham, UK | Kalyaani Persad, University of Birmingham, UK |
| 09:40 – 10:00 | Scenario-based assessment: Case 1 | Aqeelah Khatoon, University of Birmingham, UK | Nevil Philip, University Hospitals Birmingham, UK |
| 10:00 – 10:20 | Scenario-based assessment: Case 2 |  |  |
| 10:20 – 10:40 | Scenario-based assessment: Case 3 |  |  |
| 10:40 - 10:55 | Case 1 discussion | [Ketan Dhatariya, Norfolk & Norwich Hospitals, UK](https://profketandhatariya.com/) | Angelica Sharma, Norfolk and Norwich Hospitals, UK |
| 10:55 - 11:10 | Case 2 discussion | , [Gerry Rayman, Consultant in Diabetes Ipswich Hospital, UK](https://iwgdfguidelines.org/rayman/) |  |
| 11:10 - 11:25 | Case 3 discussion | [Mujahid Saeed, University Hospitals Birmingham, UK](https://mujahidsaeed.com/dr-mujahid-saeed/) |  |
| 11:25 – 11:40 | Refreshment break | | |
| 11:40 – 12:00 | Scenario-based assessment: Case 4 | Kalyaani Persad, University of Birmingham, UK | [Gerry Rayman, Consultant in Diabetes Ipswich Hospital, UK](https://iwgdfguidelines.org/rayman/) |
| 12:00 – 12:20 | Scenario-based assessment: Case 5 |  |  |
| 12:20 – 12:40 | Scenario-based assessment: Case 6 |  |  |
| 12:40 – 13:30 | Lunch | | |

| 13:30 - 13:45 | Case 4 discussion | [Sofia Salahuddin,University Hospitals Birmingham, UK](https://www.linkedin.com/in/sofia-salahuddin-60387b79/?originalSubdomain=uk) | Nevil Philip, University Hospitals Birmingham, UK |
| --- | --- | --- | --- |
| 13:45 - 14:00 | Case 5 discussion | [Srikanth Bellary, University Hospitals Birmingham, UK](https://www.linkedin.com/in/srikanth-bellary-20470a146/?originalSubdomain=uk) |  |
| 14:00 - 14:15 | Case 6 discussion | Jason Cheung, Norfolk and Norwich Hospitals, UK |  |
| 14:15 – 14:35 | Scenario-based assessment: Case 7 | Aqeelah Khatoon   University of Birmingham, UK | Angelica Sharma, Norfolk and Norwich Hospitals, UK |
| 14:35 – 14:55 | Scenario-based assessment: Case 8 |  |  |
| 14:55 – 15:15 | Scenario-based assessment: Case 9 |  |  |
| 15:15 – 15:30 | Refreshment break | | |
| 15:30 - 15:45 | Case 7 discussion | [Punith Kempegowda, University Hospitals Birmingham, UK](https://www.birmingham.ac.uk/staff/profiles/applied-health/kempegowda-punith) | [Sofia Salahuddin, Consultant in Diabetes University Hospitals Birmingham, UK](https://www.linkedin.com/in/sofia-salahuddin-60387b79/?originalSubdomain=uk) |
| 15:45 - 16:00 | Case 8 discussion | Rajeev Raghavan, Royal Wolverhampton Trust, UK |  |
| 16:00 - 16:15 | Case 9 discussion | Feaz Babwah, University Hospitals Birmingham |  |
| 16:15 – 16:45 | Technology in Acute Diabetes Care | [Mohammed Ali Karamat,University Hospitals Birmingham, UK](https://www.researchgate.net/profile/Muhammad-Karamat-2) | [Punith Kempegowda, University Hospitals Birmingham, UK](https://www.birmingham.ac.uk/staff/profiles/applied-health/kempegowda-punith) |
| 16:45 – 17:00 | Closing, Post-SIMBA survey and vote of thanks | | Kalyaani Persad, University of Birmingham, UK |

Case Transcripts Folder Available at: <https://drive.google.com/drive/folders/15_Szh1NwUsS6NWrN4b7JxCe-jGh26KnT?usp=sharing>
