# Supplementary material for: Simulation‐based training significantly improved confidence and clinical skills of resident doctors in acute diabetes management
Source: Diabet Med. 2025 Jun 17;42(9):e70068. doi: 10.1111/dme.70068 (PMC12352711; doi:10.1111/dme.70068)
Supplement: Supplementary file 6 — Data S6: [file DME-42-e70068-s004.docx]

**Supplement 6: POST SIMULATION STAKEHOLDER FEEDBACK INTERVIEW**

Section 1: Introduction

1. Introduce the interviewer/facilitator

2. Thank the participant for participating

3. Check participant’s audio and video are working

4. Confirm they are happy for us to record

START RECORDING

1. Reintroduce the interviewer/facilitator:

   - "Hi, I am ____, one of the facilitators for this research."

2. "Thank you for participating. Do you consent to the audio recording of your interview and using your pseudonymized quotes in research reports and publications?"

3. Explain the interview’s purpose:

   - "This interview aims to understand your experiences following the simulation-based learning for acute diabetes. There are no right or wrong answers, and your insights are invaluable."

4. "I will guide the conversation with questions, but feel free to share additional thoughts or let me know if a question is not relevant."

5. "You can pause the recording or leave the interview at any time."

6. "If you have any questions about data handling, please refer to the participant information sheet or contact me directly."

Section 2: Background

1. Initial experience:

   - "Could you share your overall impressions of the SIMBA session on Acute Diabetes?"

   - "What were your expectations going into the session, and did it meet those?"

2. Current training landscape:

   - "In your opinion, how does simulation-based learning fit within current training models for healthcare professionals managing acute diabetes?"

Section 3: Knowledge/Experiences

1. Realism and Alignment:

   - "How well did the simulation align with real-life scenarios you’ve encountered in acute diabetes care?"

   - "Was there any aspect of the simulation that you found particularly true to clinical practice?"

2. Key strengths of the simulation:

   - "Which elements of the simulation did you find most beneficial for learning and clinical application?"

   - "How do you think these strengths can positively influence diabetes care in practice?"

3. Areas for improvement:

   - "Are there areas of the simulation that could be enhanced to better prepare healthcare professionals for real-world acute diabetes management?"

   - "Did anything feel unrealistic or less useful for clinical practice?"

4. Resource and support needs:

   - "What additional resources or support do you believe are necessary to further equip healthcare professionals for managing acute diabetes, especially under pressure?"

Section 4: Awareness and Participation in SIMBA

1. Familiarity with SIMBA:

   - "Prior to this session, were you familiar with the SIMBA model?"

   - "If yes, what were your initial impressions of it, and how have they changed after participating in this session?"

2. Impact of SIMBA on learning:

   - "How do you see SIMBA contributing to healthcare providers' education, specifically regarding acute diabetes care?"

3. Role of simulation in future training:

   - "How can SIMBA be integrated into routine training for healthcare professionals to enhance their readiness for managing acute diabetes?"

Section 5: Effectiveness of SIMBA

1. Overall assessment:

"How would you assess the overall effectiveness of this SIMBA session in meeting the learning needs of healthcare professionals in diabetes care?"

1. Most effective components:

 "What aspects of SIMBA did you find particularly effective in enhancing understanding and management of acute diabetes?"

1. Suggestions for improvement:

"What improvements would you suggest for future SIMBA sessions, especially for diabetes care specialists?"

Section 6: Scalability and Sustainability

1. Facilitating widespread adoption:

   - "What factors do you believe would facilitate the wider adoption of simulation-based learning for acute diabetes management in healthcare organizations?"

2. Barriers and solutions:

   - "What barriers do you foresee in scaling simulation-based learning for diabetes care, and how might they be overcome?"

3. Sustainability of the model:

   - "How can simulation-based learning be sustainably integrated into healthcare education and professional development, particularly for diabetes care?"

Section 7: Networking and Collaboration

1. Networking opportunities during the session:
   - "Did the SIMBA session provide opportunities to connect and network with other professionals or experts in the field of diabetes care?"
   - "Can you share any specific examples of how the session facilitated interactions or collaboration with others?"
2. Impact on professional relationships:
   - "Did the session help you establish new professional relationships or strengthen existing ones within the diabetes care community?"
3. Collaborative learning and exchange of ideas:
   - "Did you find the session useful for sharing knowledge and exchanging ideas with peers and experts in the field?"
   - "Were there any specific interactions that helped you gain new insights or approaches to managing acute diabetes?"
4. Suggestions for enhancing networking:
   - "Do you have any suggestions for improving how SIMBA sessions could facilitate networking and collaboration among participants?"
   - "What additional features or platforms could be introduced to help build stronger professional networks through SIMBA?"

Section 8: Closing

1. Wrap-up:

   - "Is there anything we haven't covered that you feel is important to mention regarding your experience with the SIMBA session?"

   - "Do you have any final thoughts on how SIMBA can enhance acute diabetes management training?"

THANK AND FINISH RECORDING.
