# Supplementary material for: Simulation‐based training significantly improved confidence and clinical skills of resident doctors in acute diabetes management
Source: Diabet Med. 2025 Jun 17;42(9):e70068. doi: 10.1111/dme.70068 (PMC12352711; doi:10.1111/dme.70068)
Supplement: Supplementary file 7 — Data S7: [file DME-42-e70068-s006.docx]

**Supplement 7: POST SIMULATION FEEDBACK INTERVIEW WITH MODERATORS AND PARTICIPANTS**

Section 1: Introduction

1. Introduce the interviewer/facilitator

2. Thank the participant for participating

3. Check participant’s audio and video are working

4. Confirm they are happy for us to record

START RECORDING

1. Reintroduce the interviewer/facilitator:

   - "Hi, I am _____, one of the facilitators for this research."

2. "Thank you for participating. Do you consent to the audio recording of your interview and using your pseudonymized quotes in research reports and publications?"

3. Explain the interview’s purpose:

   - "This interview aims to understand your experiences with simulation-based learning for acute diabetes. There are no right or wrong answers, and your insights are invaluable."

4. "I will guide the conversation with questions, but feel free to share additional thoughts or let me know if a question is not relevant."

5. "You can pause the recording or leave the interview at any time."

6. "If you have any questions about data handling, please refer to the participant information sheet or contact me directly."

Section 2: Relevance and Expectations

1. Relevance of the content

   - "How relevant was the content of the simulation session to your current stage of training? Did it address areas you were hoping to learn more about?"

2. Pre-session expectations

   - "What were your expectations before attending the simulation? Did you have any specific goals or areas of interest related to acute diabetes management?"

3. Meeting expectations

   - "Did the session meet, exceed, or fall short of your expectations? Can you explain why?"

Section 3: Knowledge and Skills Development

1. Knowledge gained

   - "How has your understanding of acute diabetes management changed after attending the session? What key takeaways or new information did you learn?"

2. Specific skills or competencies

   - "Can you share specific examples of skills or knowledge that you improved or developed through the simulation? How do you think these will help you in clinical practice?"

3. Problem-solving and decision-making

   - "How did the simulation challenge your problem-solving skills or decision-making, especially in high-pressure or urgent situations? Could you describe a specific scenario from the session?"

4. Emotional and cognitive reactions

   - "Did the simulation provoke any strong emotional or cognitive responses? How did it feel to be in those situations, and what did you learn from it?"

Section 4: Collaboration and Team Dynamics

1. Teamwork and collaboration

   - "How effective was the simulation in fostering collaboration or teamwork? Did you feel supported by the group, and how did it influence your learning?"

2. Improvements in team-based learning

   - "Is there anything that could be improved in the way the simulation facilitates team-based problem-solving or decision-making?"

Section 5: Learning Gaps and Challenges

1. Learning gaps or unaddressed topics

   - "Were there any specific topics or areas related to acute diabetes management that you feel were underrepresented in the session?"

2. Unexpected challenges

   - "What aspects of the simulation surprised you or differed from what you expected going in? Were there any unexpected challenges that you faced during the session?"

Section 6: Application to Real-World Practice

1. Confidence in applying knowledge

   - "How confident do you feel about applying the skills or knowledge you gained from the simulation to real-world inpatient settings? Why or why not?"

2. Practicality of the simulation

   - "How well did the simulation prepare you for real-life scenarios involving acute diabetes management? Can you think of a particular aspect of the training that will be immediately useful in your clinical practice?"

Section 7: Overall Experience and Feedback

1. Overall experience

   - "How would you describe your overall experience with the simulation? What were the most impactful aspects of the session?"

2. Suggestions for improvement

   - "Is there anything that could be improved in future simulations to enhance learning for students and resident doctors?"

3. Future expectations

   - "Looking ahead, what additional support or resources do you think would be beneficial for further training in managing acute diabetes?"

THANK AND STOP RECORDING.
