# Supplementary material for: Simulation‐based training significantly improved confidence and clinical skills of resident doctors in acute diabetes management
Source: Diabet Med. 2025 Jun 17;42(9):e70068. doi: 10.1111/dme.70068 (PMC12352711; doi:10.1111/dme.70068)
Supplement: Supplementary file 8 — Data S8: [file DME-42-e70068-s009.docx]

**Supplement 8: Themes and Domains developed from post-session interviews with Medical Students and Resident Doctors**

**Medical Students and Resident Doctors**

Kappa Coefficient with Child Codes – 0.60

Kappa Coefficient without Child Codes – 0.52

| **Code** | **Theme** | **Domain** |
| --- | --- | --- |
| Challenges of the simulation faced by medical students | Good representation of areas in acute diabetes management during the session | Enhanced Understanding and Confidence in Acute Diabetes Management (n=219) |
| Advanced planning | Knowledge developed through simulation |  |
| Blood glucose monitoring |  |  |
| Guidelines |  |  |
| Technological advancements |  |  |
| TPN feed |  |  |
| VRII-induced hyponatraemia |  |  |
| Improved understanding of acute diabetes | Improvement in understanding of acute diabetes management |  |
| Improved understanding of acute diabetes management post-surgery |  |  |
| Improved understanding of diabetic foot disease |  |  |
| Improved understanding of DKA or HHS |  |  |
| Improved understanding of palliative care or end of life care in acute diabetes |  |  |
| Improved understanding of steroid induced hyperglycaemia |  |  |
| Improved understanding of VRII-induced hyponatraemia |  |  |
|  | Increased confidence in applying skills or knowledge to real-world scenarios |  |
| Acute vs Long-term management | Additional support or resources on acute diabetes management |  |
| Case-based learning |  |  |
| Enhanced guideline awareness |  |  |
| Interactive pathway |  |  |
| Simulation-based assessment |  |  |
| Step-wise approach |  |  |
| Teaching sessions for FY |  |  |
| Antenatal.Prenatal diabetic care | Areas of acute diabetes underrepresented during the session | Challenges and Limitations of Simulation-Based Learning (n=75) |
| Diabetic remission |  |  |
| Hypoglycaemia |  |  |
| Iatrogenic diabetes besides steroids |  |  |
| Perioperative diabetic care |  |  |
| Technological advancements |  |  |
| Cross-specialty management | Challenges of the simulation faced by resident doctors |  |
| Technical difficulties |  |  |
| Treat simulation seriously |  |  |
| Navigation skills | Challenges of the simulation faced by medical students |  |
| Poor virtual experience |  |  |
| Targeted needs |  |  |
| Yes | Emotional or cognitive response provoked by the simulation |  |
| No |  |  |
| High relevance to medical student | Relevance of the session to their stage of training | Role of Simulation in Developing Clinical and Problem-Solving Skills (n=146) |
| High relevance to resident doctors |  |  |
| High relevance for medical educationist |  |  |
| Advanced care planning | Aspects of simulation that will be useful in clinical practice |  |
| Guideline signposting |  |  |
| Importance of monitoring in **persons with diabetes** |  |  |
| Insulin prescription in DKA |  |  |
| Making sure to check feet in a **persons with diabetes** |  |  |
| Making sure to follow a clear structure when interacting with patients |  |  |
| Work well under time pressure |  |  |
| All-around awareness | Skills developed through simulation |  |
| Application utilisation - Whatsapp and Word |  |  |
| Communication.Networking skills |  |  |
| Critical thinking skills |  |  |
| Expert-participant interactions |  |  |
| Holistic care |  |  |
| Learning to think as a doctor |  |  |
| Listening skills |  |  |
| MDT involvement |  |  |
| Organisation or teamworking skills developed through simulation |  |  |
| Presentation skills |  |  |
| Relevant investigations |  |  |
| Teaching and feedback provision |  |  |
| Timekeeping skill |  |  |
|  | Problem-solving or decision-making skills developed during the session |  |
|  | Overall experience of session | Feedback and Suggestions for Improvement of Simulation Sessions (n=295) |
|  | Expectations before attending the simulation |  |
| DKA and its management | Specific goals or areas of interest before attending simulation |  |
| HHS and its management |  |  |
| Hypoglycaemia and its management |  |  |
|  | Simulation met the person's expectations |  |
|  | Simulation exceeded the person's expectations |  |
| Comprehensive training | What worked well in the session |  |
| Emulation of the real-life situation |  |  |
| Expert discussions were a highlight of the session |  |  |
| Good organisation or logistics of the session |  |  |
| Guideline provision |  |  |
| Introductory video |  |  |
| Model answer provision |  |  |
| Networking opportunity |  |  |
| Quality and complexity of cases |  |  |
| Real-time messaging |  |  |
| Simulation-based learning |  |  |
| Structural approach |  |  |
| Sufficient breaks |  |  |
| Suitable learning environment |  |  |
| Application of SIMBA to other specialties | Suggested improvements for future sessions |  |
| Changing the format or structure of simulations |  |  |
| Check-up on attendance |  |  |
| Clear target audience |  |  |
| Forms checking |  |  |
| Group discussions |  |  |
| Improvement for virtual participants |  |  |
| Increased frequency |  |  |
| Information provision |  |  |
| Moderator training |  |  |
| Potential for recording |  |  |
| Reminders on electronic devices |  |  |
| Summary of learning points |  |  |
| Transcript proofreading and improvement |  |  |
| Lack of teamwork or collaboration | Teamwork or collaboration during the simulation |  |
| Teamwork present during the simulation |  |  |
|  | Suggestions on how to improve teamwork or collaboration during the simulation |  |

1. **Medical students**

Kappa coefficient with child codes = 0.59

| Code | Theme | Domain |
| --- | --- | --- |
| Challenges of the simulation faced by medical students | Good representation of areas in acute diabetes management during the session | Enhanced Understanding and Confidence in Acute Diabetes Management (n=121) |
| Advanced planning | Knowledge developed through simulation |  |
| Blood glucose monitoring |  |  |
| Guidelines |  |  |
| Knowledge developed on acute diabetes Guidelines |  |  |
| Knowledge developed on acute diabetes management |  |  |
| Technological advancements |  |  |
| TPN feed |  |  |
| Improved understanding of acute diabetes | Improvement in understanding of acute diabetes management |  |
| Improved understanding of acute diabetes management post-surgery |  |  |
| Improved understanding of diabetic foot disease |  |  |
| Improved understanding of DKA or HHS |  |  |
| Improved understanding of palliative care or end of life care in acute diabetes |  |  |
| Improved understanding of steroid induced hyperglycaemia |  |  |
|  | Increased confidence in applying skills or knowledge to real-world scenarios |  |
| Case-based learning | Additional support or resources on acute diabetes management |  |
| Enhanced guideline awareness |  |  |
| Interactive pathway |  |  |
| Teaching sessions for FY |  |  |
| Navigation skills | Challenges of the simulation faced by medical students | Challenges and Limitations of Simulation-Based Learning (n=32) |
| Poor virtual experience |  |  |
| Targeted needs |  |  |
| Yes | Emotional or cognitive response provoked by the simulation |  |
| No |  |  |
| High relevance to medical student | Relevance of the session to their stage of training | Role of Simulation in Developing Clinical and Problem-Solving Skills (n=86) |
| High relevance to resident doctors |  |  |
| Importance of monitoring in diabetic patients | Aspects of simulation that will be useful in clinical practice |  |
| Insulin prescription in DKA |  |  |
| Making sure to check feet in a diabetic patient |  |  |
| Making sure to follow a clear structure when interacting with patients |  |  |
| All-around awareness | Skills developed through simulation |  |
| Application utilisation - Whatsapp and Word |  |  |
| Communication.Networking skills |  |  |
| Critical thinking skills |  |  |
| Holistic care |  |  |
| Learning to think as a doctor |  |  |
| Listening skills |  |  |
| Organisation or teamworking skills developed through simulation |  |  |
| Presentation skills |  |  |
| Teaching and feedback provision |  |  |
| Timekeeping skill |  |  |
|  | Problem-solving or decision-making skills developed during the session |  |
|  | Overall experience of session | Feedback and Suggestions for Improvement of Simulation Sessions (n=146) |
|  | Expectations before attending the simulation |  |
| HHS and its management | Specific goals or areas of interest before attending simulation |  |
|  | Simulation met the person's expectations |  |
|  | Simulation exceeded the person's expectations |  |
| Comprehensive training | What worked well in the session |  |
| Emulation of the real-life situation |  |  |
| Expert discussions were a highlight of the session |  |  |
| Good organisation or logistics of the session |  |  |
| Networking opportunity |  |  |
| Structural approach |  |  |
| Suitable learning environment |  |  |
| Changing the format or structure of simulations | Suggested improvements for future sessions |  |
| Check-up on attendance |  |  |
| Clear target audience |  |  |
| Improvement for virtual participants |  |  |
| Information provision |  |  |
| Moderator training |  |  |
| Transcript proofreading and improvement |  |  |
| Teamwork present during the simulation | Teamwork or collaboration during the simulation |  |
|  | Suggestions on how to improve teamwork or collaboration during the simulation |  |

1. **Resident Doctors**

Kappa coefficient with child codes = 0.62

| Code | Theme | Domain |
| --- | --- | --- |
|  | Good representation of areas in acute diabetes management during the session | Enhanced Understanding and Confidence in Acute Diabetes Management (n=98) |
| Advanced planning | Knowledge developed through simulation |  |
| Blood glucose monitoring |  |  |
| Guidelines |  |  |
| Technological advancements |  |  |
| TPN feed |  |  |
| VRII-induced hyponatraemia |  |  |
| Improved understanding of acute diabetes | Improvement in understanding of acute diabetes management |  |
| Improved understanding of acute diabetes management post-surgery |  |  |
| Improved understanding of diabetic foot disease |  |  |
| Improved understanding of DKA or HHS |  |  |
| Improved understanding of palliative care or end of life care in acute diabetes |  |  |
| Improved understanding of steroid induced hyperglycaemia |  |  |
| Improved understanding of VRII-induced hyponatraemia |  |  |
|  | Increased confidence in applying skills or knowledge to real-world scenarios |  |
| Acute vs Long-term management | Additional support or resources on acute diabetes management |  |
| Simulation-based assessment |  |  |
| Step-wise approach |  |  |
| Antenatal.Prenatal diabetic care | Areas of acute diabetes underrepresented during the session | Challenges and Limitations of Simulation-Based Learning (n=43) |
| Diabetic remission |  |  |
| Hypoglycaemia |  |  |
| Iatrogenic diabetes besides steroids |  |  |
| Perioperative diabetic care |  |  |
| Technological advancements |  |  |
| Cross-specialty management | Challenges of the simulation faced by resident doctors |  |
| Technical difficulties |  |  |
| Treat simulation seriously |  |  |
| Yes | Emotional or cognitive response provoked by the simulation |  |
| No |  |  |
| High relevance to medical student | Relevance of the session to their stage of training | Role of Simulation in Developing Clinical and Problem-Solving Skills (n=60) |
| High relevance to resident doctors |  |  |
| High relevance for medical educationist |  |  |
| Advanced care planning | Aspects of simulation that will be useful in clinical practice |  |
| Guideline signposting |  |  |
| Making sure to check feet in a diabetic patient |  |  |
| Work well under time pressure |  |  |
| Critical thinking skills | Skills developed through simulation |  |
| Expert-participant interactions |  |  |
| Holistic care |  |  |
| MDT involvement |  |  |
| Relevant investigations |  |  |
|  | Problem-solving or decision-making skills developed during the session |  |
|  | Overall experience of session | Feedback and Suggestions for Improvement of Simulation Sessions (n=149) |
|  | Expectations before attending the simulation |  |
| DKA and its management | Specific goals or areas of interest before attending simulation |  |
| HHS and its management |  |  |
| Hypoglycaemia and its management |  |  |
|  | Simulation met the person's expectations |  |
|  | Simulation exceeded the person's expectations |  |
| Comprehensive training | What worked well in the session |  |
| Emulation of the real-life situation |  |  |
| Expert discussions were a highlight of the session |  |  |
| Good organisation or logistics of the session |  |  |
| Guideline provision |  |  |
| Introductory video |  |  |
| Model answer provision |  |  |
| Networking opportunity |  |  |
| Quality and complexity of cases |  |  |
| Real-time messaging |  |  |
| Simulation-based learning |  |  |
| Sufficient breaks |  |  |
| Application of SIMBA to other specialties | Suggested improvements for future sessions |  |
| Changing the format or structure of simulations |  |  |
| Forms checking |  |  |
| Group discussions |  |  |
| Improvement for virtual participants |  |  |
| Increased frequency |  |  |
| Information provision |  |  |
| Potential for recording |  |  |
| Reminders on electronic devices |  |  |
| Summary of learning points |  |  |
| Transcript proofreading and improvement |  |  |
| Lack of teamwork or collaboration | Teamwork or collaboration during the simulation |  |
| Teamwork present during the simulation |  |  |
|  | Suggestions on how to improve teamwork or collaboration during the simulation |  |
