# Supplementary material for: Simulation‐based training significantly improved confidence and clinical skills of resident doctors in acute diabetes management
Source: Diabet Med. 2025 Jun 17;42(9):e70068. doi: 10.1111/dme.70068 (PMC12352711; doi:10.1111/dme.70068)
Supplement: Supplementary file 9 — Data S9: [file DME-42-e70068-s007.docx]

**Supplement 9: Themes and Domains developed from post-session interviews with Stakeholders**

Kappa coefficient = 0.64

| Code(s) | Theme(s) | Domain(s) |
| --- | --- | --- |
| Beneficial aspects of simulation | Benefits of Simulation-Based Learning | Benefits and Challenges of Simulation-Based Learning (n=119) |
| Benefits of simulation-based learning for training in acute diabetes management |  |  |
| Positive or effective aspects of SIMBA for acute diabetes management |  |  |
| SIMBA’s contribution to educating on acute diabetes care |  |  |
| Barriers for scaling simulation-based learning for acute diabetes care | Challenges of Simulation-Based Learning |  |
| Aspects of the simulation that did not align with real-life acute diabetes scenarios, or felt unrealistic or less useful for clinical practice |  |  |
| No | Previous experience with SIMBA model | Improving Simulation Engagement and Realism (n=156) |
| Yes |  |  |
| More interaction from the beginning | Areas of improvement for simulation |  |
| More opportunities to undertake simulation |  |  |
| Tailor to audience, either medical student or SPR |  |  |
| Encourage mixing between consultants and resident doctors or medical students | Improvements for future SIMBA sessions on diabetes |  |
| Encourage mixing between consultants and resident doctors/medical students |  |  |
| Encourage students to ask questions |  |  |
| Incentivize experts using small monetary charge for participants |  |  |
| Make it interactive from the beginning |  |  |
| More time for discussion |  |  |
| More time on each case |  |  |
|  | Expectations before attending session |  |
| Yes | Expectations of session met |  |
| No |  |  |
|  | Initial impressions of SIMBA and how they’ve changed after session |  |
| Decision making | Aspects of simulation that were well aligned with real-life acute diabetes scenarios |  |
| HHS scenario |  |  |
| Principles of management |  |  |
| Uncertainty |  |  |
| Guideline App | Additional support or resources for managing acute diabetes |  |
| QR code to guidelines |  |  |
| Simple flow chart |  |  |
| Engaging | Overall impression of session |  |
| Enjoyable |  |  |
| Interactive |  |  |
| Well organised |  |  |
| Accessibility | Facilitators for adopting simulation-based learning within training for acute diabetes management | Facilitating Adoption and Integration of SIMBA in Training (n=111) |
| Dedicated curriculum time |  |  |
| Increased awareness |  |  |
| Word of mouth |  |  |
| Incorporate into formal training induction | How to integrate SIMBA into routine training for acute diabetes |  |
| Integrate into registrar training programme |  |  |
| Make time within curriculum |  |  |
| Regular sessions every 6 months |  |  |
| Increase awareness through national forum | Suggestions to sustainably integrate simulation-based learning into healthcare education for acute diabetes |  |
| More curriculum time |  |  |
| More funding |  |  |
| Record sessions to increase convenience |  |  |
| ABCD | Professional platforms that would be useful |  |
| Avoid twitter |  |  |
| Diabetes update |  |  |
| Use Linkedin |  |  |
| YDEF |  |  |
| Opportunity to catch up on publications | Networking or collaboration opportunities provided by SIMBA session | Networking and Professional Collaboration (n=86) |
| Opportunity to catch up with collegues |  |  |
| Helped to establish new relationships | Effect of session on professional relationships |  |
| Strengthened existing relationships |  |  |
| No | Session was useful for sharing knowledge or exchanging ideas with peers and experts in the field |  |
| Yes |  |  |
| Discussion on early detection of T1D | Specific interactions that provided new insights into acute diabetes management |  |
| Discussions on inpatient diabetes |  |  |
| Discussions on service development |  |  |
| Role of technology in an inpatient setting |  |  |
| SGLT2 inhibitors debate |  |  |
| Encourage socialisation between consultants and medical students/junior doctors | Suggestions on how to improve networking and collaboration through SIMBA |  |
| Encourage socialisation between consultants and medical students/resident doctors |  |  |
